# Severe ophthalmic manifestation in pituitary-involved granulomatosis with polyangiitis: a case report and literature review

**DOI:** 10.1186/s12886-018-0966-0

**Published:** 2018-11-16

**Authors:** Xia Zhang, Bing Xing, Hui You, Huanwen Wu, Yong Zhong, Jin Ma

**Affiliations:** 1 Department of Ophthalmology, Peking Union Medical College Hospital, Chinese Academy of Medical Sciences and Peking Union Medical College, No. 1 Shuaifuyuan Wangfujing Dongcheng District, Beijing, China; 2 Department of Neurosurgery, Peking Union Medical College Hospital, Chinese Academy of Medical Sciences and Peking Union Medical College, No. 1 Shuaifuyuan Wangfujing Dongcheng District, Beijing, China; 3 Department of Radiology, Peking Union Medical College Hospital, Chinese Academy of Medical Sciences and Peking Union Medical College, No. 1 Shuaifuyuan Wangfujing Dongcheng District, Beijing, China; 4 Department of Pathology, Peking Union Medical College Hospital, Chinese Academy of Medical Sciences and Peking Union Medical College, No. 1 Shuaifuyuan Wangfujing Dongcheng District, Beijing, China

**Keywords:** Pituitary, Granulomatosis with polyangiitis, ANCA

## Abstract

**Background:**

Granulomatosis with polyangiitis (GPA), a necrotizing granulomatous disease, very rarely involves the central nervous system (CNS), particularly the pituitary. Delayed treatment may cause permanent bilateral blindness. We report an isolated case of pituitary GPA that manifested as a progressive bilateral temporal visual field (VF) defect and was diagnosed via pituitary biopsy. Additionally, we review ocular, chiasmal and cranial nerve involvement in pituitary GPA.

**Case presentation:**

A 20-year-old Chinese man was referred for repeated fever, sudden headache, diplopia with a bilateral best-corrected visual acuity (BCVA) of 10/20, ptosis in both eyes and restricted abduction on the right side. VF tests showed bitemporal hemianopsia. Laboratory tests revealed hypothyroidism and were negative for autoimmune markers. Enhanced magnetic resonance imaging (MRI) showed pituitary enlargement. The diagnosis was lymphocytic pituitaritis. After intravenous (IV) dexamethasone treatment, full recovery occurred within 2 months. Two years later, the patient was readmitted for headache recurrence. With oral prednisone, the visual acuity in his right eye rapidly decreased to hand motion (HM) within one month. Enhanced MRI showed pituitary enlargement and a new, invasive suprasellar CNS lesion. All infection- and autoimmune-related tests were negative. The visual acuity in his right and left eye decreased to no light perception (NLP) after 6 days and 2 weeks, respectively. The biopsy results suggested GPA. After IV methylprednisolone treatment, complete remission of the symptoms occurred and was confirmed by MRI. The 15-month follow-up showed no signs of recurrence.

**Conclusion:**

GPA typically affects the respiratory tract, lungs and kidneys. To date, 50 cases with pituitary involvement have been reported. Chiasmal and cranial nerve involvement leading to visual acuity impairment are common. We found 2 cases with severe visual loss resembling our case and discuss certain similarities.

**Electronic supplementary material:**

The online version of this article (10.1186/s12886-018-0966-0) contains supplementary material, which is available to authorized users.

## Background

Granulomatosis with polyangiitis (GPA), formerly known as Wegener’s granulomatosis (WG), is an anti-neutrophil cytoplasmic antibody (ANCA)-related necrotizing granulomatous disease. GPA typically affects the upper respiratory tract, lungs and kidneys, but all organs can be involved because the disease is a systemic form of granulomatous small-vessel vasculitis. The central nervous system (CNS), particularly the pituitary, is rarely involved [1]. Until 2016, only 2 cases of GPA with isolated pituitary-involvement had been reported in the English literature. Currently, the diagnosis of GPA depends solely on clinical symptoms. While pathological biopsy is the gold standard for diagnosing GPA, atypical cases are diagnosed with the assistance of serological examinations. The early diagnosis of GPA is important for the treatment and prognosis of this disease, especially when the chiasm and optic nerve are involved. Herein, we report an isolated case of pituitary GPA that manifested as a progressive bilateral temporal visual field (VF) defect, was diagnosed by pituitary biopsy and rapidly developed to bilateral blindness; in addition, we review ocular, chiasmal and cranial nerve involvement in pituitary GPA.

## Case presentation

**Fig. 1:**
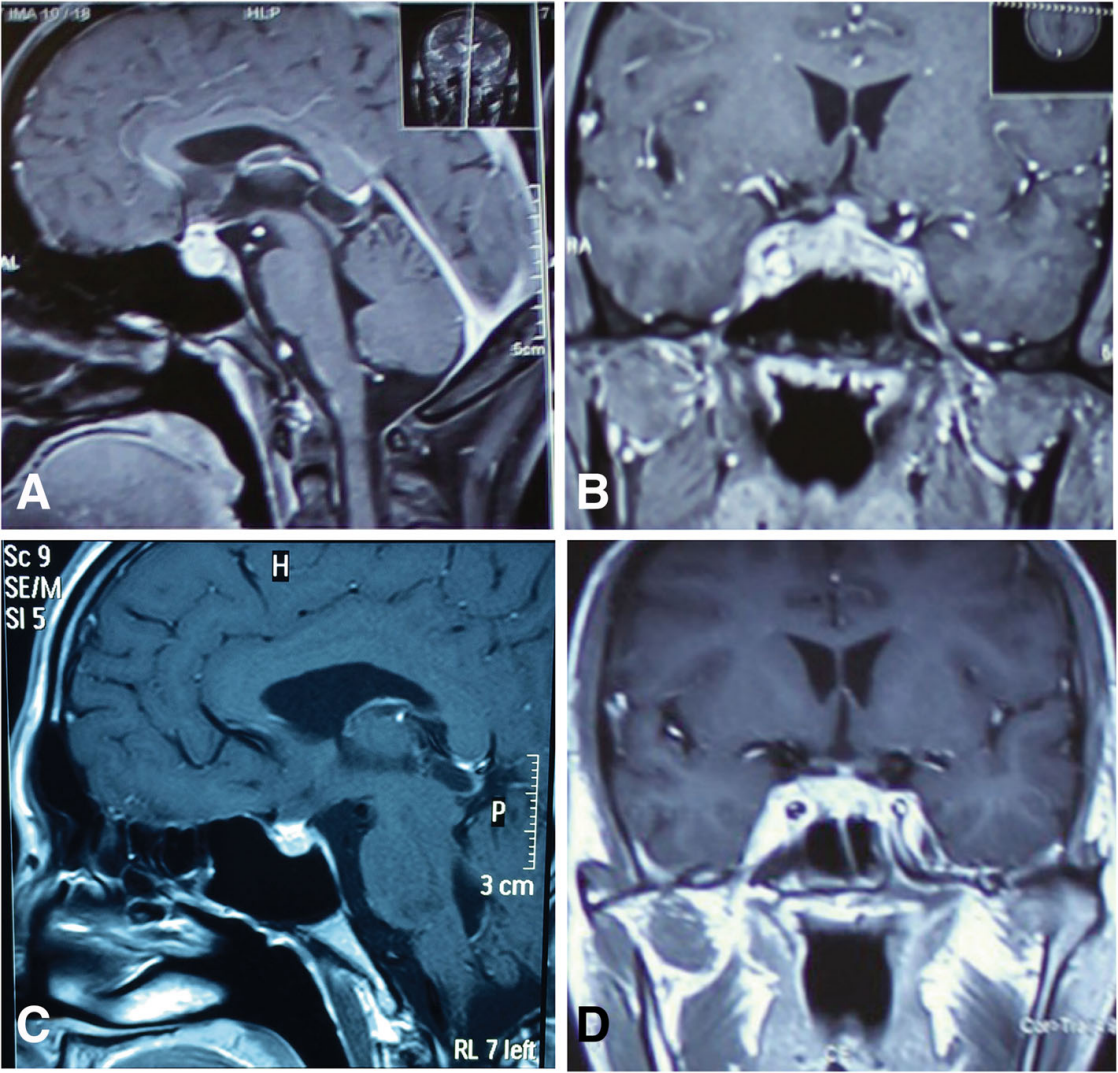
Magnetic resonance imaging (MRI) scans from the first episode: **a** Enhanced sagittal T1 sequence: enlarged pituitary fossa with a lesion convex to the suprasellar area. The pituitary stalk was compressed. The size of the lesion was 43*15*17 mm, with a slightly long T1 and long T2 signal mixed with a short T1 signal and a long T2 signal with inhomogeneous enhancement. No obvious bone destruction was detected. **b** Enhanced coronal T1 sequence: optic chiasm compression with the bilateral cavernous sinus surrounded by the lesion. **c** Enhanced sagittal T1 sequence: the volume of the lesion was reduced significantly after IV glucocorticoid treatment. **d** Enhanced coronal T1 sequence: the morphology of the optic chiasm and cavernous sinus returned to normal after IV glucocorticoid administration

A 20-year-old Chinese man with no significant medical history was referred for sudden headache with diplopia. His sudden headache started in July 2012 and was aggravated over 3 months by fatigue, recurrent fever, nausea and weight loss, followed by bilateral vision loss and intermittent diplopia. His body temperature was repeatedly elevated, with a maximum temperature of 39.7 °C. His best-corrected visual acuity (BCVA) was 10/20 bilaterally, with a normal intraocular pressure (IOP). He showed ptosis in both eyes, with restricted abduction on the right side. A slit-lamp examination yielded normal results for both the anterior segment and the fundus, with no relative afferent pupillary defect (RAPD). VF testing revealed bitemporal hemianopsia. Laboratory tests showed a white blood cell (WBC) count of 6.26 × 10^9^/L and a neutrophil count of 3.73 × 10^9^/L (59.5%). His renal function was normal, with a creatinine (Cr) and urea level of 61.17 μmol/L and 4.05 mmol/L, respectively. The urine was negative for protein and red blood cells. The urine-specific gravity was normal, while endocrine tests revealed a thyroid-stimulating hormone (TSH) level of 0.04 μIU/mL, an adrenocorticotropic hormone (ACTH) level of 1.70 pg/mL and a testosterone level of < 20.0 pg/mL. Morning cortisol, prolactin (PRL), random blood glucose and glycosylated hemoglobin levels were normal (Table 1). Enhanced MRI showed pituitary enlargement with increased T2 signal intensity and heterogeneous enhancement. The sellar mass displayed a suprasellar extension and optic chiasm compression, along with bilateral extension into the cavernous sinus (Fig. 1a and b). No abnormalities were found by chest or abdominal computed tomography (CT) or in the levels of tumor markers, C-reactive protein (CRP), antistreptolysin O (ASO) or rheumatoid factor (RF). The immune test results were negative for ANCAs (myeloperoxidase [MPO]-ANCAs, 3.89 RU/mL; proteinase 3 (PR3)-ANCAs, 3.09 RU/mL; reference interval, < 20 RU/mL), as well as antinuclear antibodies (ANAs) and anti-extractable nuclear antigen (ENA) antibodies. The total serum IgG level was 12.30 g/L (7.00–17.00), with an IgA level of 2.01 g/L (0.70–4.00) and an IgM level of 0.35 g/L (0.40–2.30). The patient was suspected to have immune-related pituitaritis. The cerebral spinal fluid (CSF) was then tested. The results indicated a WBC count of 13*10^6^/L and an IgG level of 4.63 mg/dL in the CSF. IgG oligoclonal bands were absent in the serum and CSF, which had no traces of bacteria, such as *Staphylococcus aureus* and *Mycobacterium tuberculosis*. A CSF smear showed a mass of lympho-monocytes and macrophages. The patient was then diagnosed with lymphocytic hypophysitis and was treated with IV dexamethasone (20 mg qd) for 3 days, followed by a decreased dosage of dexamethasone (10 mg qd*7 days, 5 mg qd*2 days) and then oral prednisone (60 mg qd). Two months later, the patient’s BCVA recovered to 100/100 bilaterally with a normal VF. His ocular movement was normal, and he reported no diplopia or headache. Additionally, the endocrine hormone levels were within normal limits (Table 1). A repeat enhanced MRI showed that the pituitary mass was smaller than before with homogeneous enhancement, and the chiasmal compression had diminished (Fig. 1c and d). The patient’s condition remained stable during the following year, with no significant changes observed by MRI.

**Table 1:** Pituitary hormone levels

	First onset	First decrease	Recurrence	Recurrence (treated)	Reference
ACTH pg/mL	1.70	NA	46.70	< 5	0–46
TSH, μIU/mL	0.04	0.95	0.67	2.10	0.38–4.34
fT4, ng/dL	0.83	0.92	0.79	1.16	0.81–1.89
FSH, mIU/mL	4.30	8.06	7.49	12.29	1.27–19.26
LH, mIU/mL	0.88	3.27	4.93	6.34	1.24–8.62
E2, pg/mL	< 20.00	603.89 ng/dL	3.33	3.08 ng/mL	1.75–7.81
PRL, ng/mL	7.42	19.50	16.39	12.41	2.64–13.13
Morning cortisol, μg/dL	0.30	2.48	10.69	5.95	4.0–22.3
Random blood glucose mmol/L	4.76	4.10	4.52	4.58	3.9–6.1
GH ng/mL	NA	NA	0.79	0.9	< 2.0
IGF-1 ng/mL	NA	NA	257	251	116–358

In Sept. 2014, he was readmitted to the local hospital due to headache recurrence with nausea and vomiting. Endocrine tests showed an elevated PRL level and hypothyroidism (Table 1). Repeated serum immune tests yielded negative results for ANAs, MPO-ANCAs and PR3-ANCAs. Enhanced MRI revealed pituitary enlargement with stalk compression and chiasmal thickening (Fig. 4a), indicating recurrent lymphocytic pituitaritis, which was treated with oral corticoids (60 mg qd). The endocrine hormone levels returned to normal, but the headache was not relieved.

**Fig. 2:**
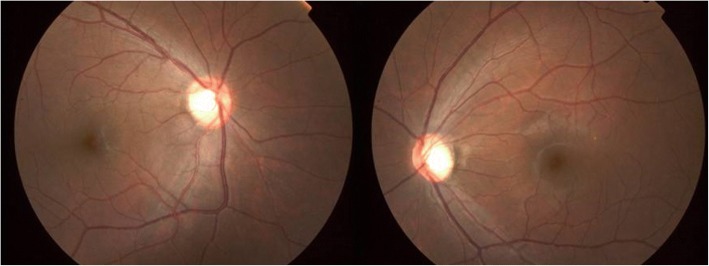
Image of the fundus at recurrence (Oct. 2014) showing a pale area in the temporal portion of the bilateral optic discs, with an obvious nerve fiber layer defect

**Fig. 3:**
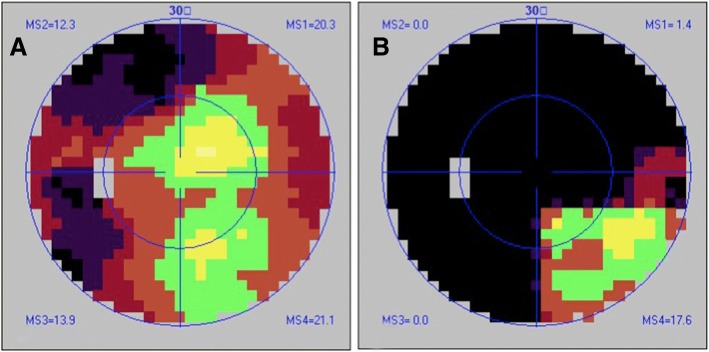
Visual field changes during disease recurrence. **a** Temporal hemianopsia was detected at the beginning of recurrence (Oct. 22, 2014). Left best-corrected visual acuity (BCVA), 20/25. **b** As the disease progressed (Oct. 31, 2014), temporal hemianopsia and supranasal quadrantanopia were detected. Left BCVA, hand motion (HM)

**Fig. 4:**
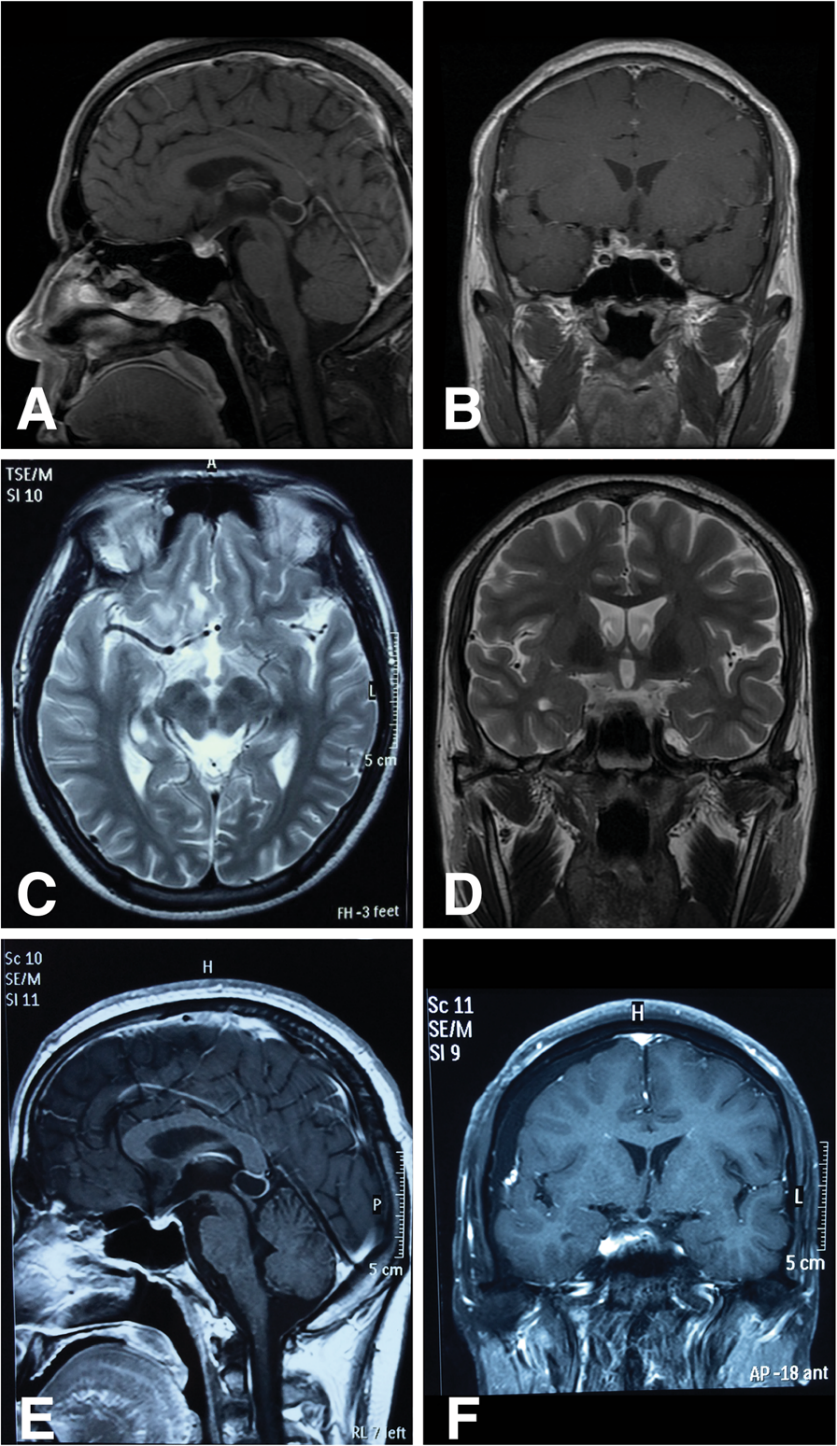
Magnetic resonance imaging (MRI) scans at recurrence and after biopsy. **a** Enhanced sagittal T1 sequence: scans after the recurrence of headache in Sept. 2014 showed an enlarged sella with a slightly sunken bottom. The pituitary was enlarged with a height of 1.15 cm and showed a heterogeneous signal, with a patchy, short T1 signal and an abnormal, long T2 signal. The pituitary stalk was shortened and thickened. **b** Enhanced coronal T1 sequence: a new lesion was detected after recurrence. There was nodular enhancement with abnormal T1 and T2 signals on the right side of the suprasellar region; the lesion was invading the pituitary stalk, infundibulum, right optic nerve, posterior right basal gyrus rectus of the frontal lobe, anterior perforated substance and extending to the internal carotid artery. **c** and **d** Enhanced T2 sequence: cerebral parenchymal edema was detected around the lesion. **e** Enhanced sagittal T1 sequence: changes were observed in the pituitary after the biopsy and i.v. administration of glucocorticoids. **f** Enhanced coronal T1 sequence: after the biopsy and i.v. administration of glucocorticoids, the parenchymal edema was significantly reduced

In Oct. 2014, the patient’s headache worsened with severe nausea and vomiting, and the visual acuity in his right eye decreased to hand motion (HM), with 80/100 in the left eye and an IOP of 13/17 mmHg. His left eye displayed ptosis, but the ocular position and eye movement were normal. A slit-lamp examination showed no abnormal findings in the anterior chamber, with an equal pupil size, but the right eye was RAPD positive. The fundus examination was normal except for bilateral pale optic papillomas (Fig. 2). VF testing revealed total blindness in the right eye and temporal hemianopsia in the left eye (Fig. 3a). Optical coherence tomography (OCT) showed a significant decrease in the thickness of the retinal nerve fiber layer. Repeated enhanced MRI showed pituitary enlargement and a new CNS lesion with abnormal nodal T1 and T2 enhancement on the right side of the suprasellar region; the lesion was invading the pituitary stalk, infundibulum, right optic nerve, posterior right basal gyrus rectus of the frontal lobe, and anterior perforated substance and extending to the internal carotid artery (Fig. 4b).

Erythrocyte sedimentation rate (ESR), CRP, cryptococcal antigen, serum 1,3-beta-D-glucan assay (BDG test), interferon gamma release assay for tubuerculosis (T-SPOT test) and a lymphocyte culture yielded negative results. An enhanced paranasal CT scan showed only bilateral ethmoid and left sphenoid sinus inflammation. No positive results were detected by chest X-ray or multiple-organ B-mode ultrasound examination. Moreover, there were no traces of red blood cells or protein in his urine. The patient’s renal function was also normal (urea, 5.03 mmol/L; Cr, 58.04 μmol/L). A multidisciplinary consultation concluded that with 30 days of oral corticosteroid therapy and no signs of relief, a CNS infection should not be excluded; therefore, oral prednisone (35 mg qd) was continued. The visual acuity of the patient’s right eye decreased to NLP 6 days later, with headache aggravation, sudden nausea and vomiting, and a reduction in the visual acuity of the left eye to counting fingers (CF). An ophthalmological examination, including an assessment of eye position and movement and the anterior and posterior segments, yielded the same results as before. Repeated VF test showed a temporal hemifield and a superior nasal quadrant defect (Fig. 3b). Repeated enhanced MRI showed meningeal linear enhancement. Two days later, the visual acuity in his left eye decreased to NLP with bilateral pupil mydriasis and disappearance of the light reflex. The results of a biopsy conducted in Dec. 2014 suggested GPA (see the Histopathology section). The patient was treated with IV methylprednisolone (500 mg qd for 3 days, followed by 250 mg qd for 3 days and then 125 mg qd for 3 days). He claimed complete headache and left proptosis remission but showed no improvement in the bilateral visual acuity or pupil reflex. MRI showed a significant reduction in the parenchymal and chiasmal edema (Fig. 4e and f). The patient’s pituitary biopsy confirmed the pathological manifestation, but repeated tests showed negative results regarding hematuria, proteinuria, and renal function and no abnormalities on chest X-ray or paranasal sinus CT. He did meet one of the 1990 American College of Rheumatology (ACR) GPA diagnostic criteria. However, considering his biopsy results and excellent response to corticosteroid therapy, we considered the diagnosis to be GPA with isolated pituitary involvement. The patient and his family requested the cessation of treatment and refused immunosuppressive therapy. 15 months after treatment with oral prednisone starting at 60 mg qd and decreasing by 5 mg every two weeks, he showed no signs of recurrence (May 2016).

### Histopathology examination

**Fig. 5:**
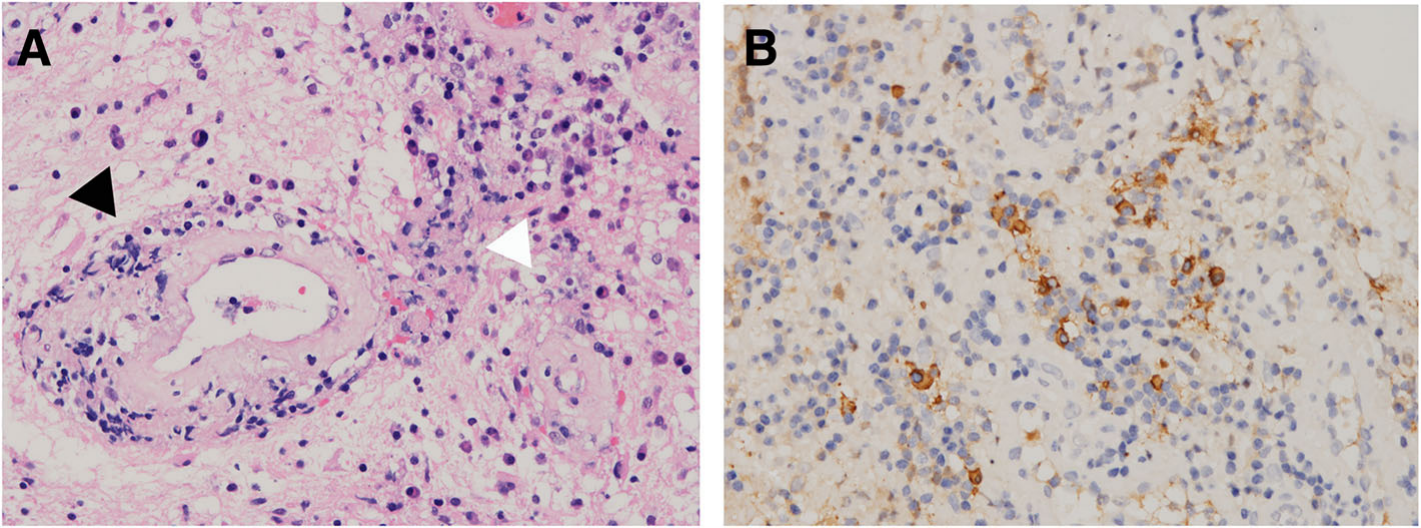
**a** Hematoxylin and eosin (H&E) staining showing a normal arrangement of acinar cells with scattered Langerhans cells, giant cells, and a large number of lymphocytes and plasma cells (white arrow), indicating granulomatous inflammation. Small blood vessels showed fibrinoid necrosis with neutrophilic and lymphocytic infiltration (black arrow) (H&E staining 40x). **b** IgG4 staining showed a positive rate of approximately 20% (IgG4 immunohistochemical staining, 40x)

The pituitary tissue specimen was stained and tested at two institutions in China (Sanbo Neurology Hospital and Peking Union Medical College Hospital), with similar results. Repeated acid-fast staining showed negative results. Hematoxylin and eosin (H&E) staining (Fig. 5a) showed a normal arrangement of acinar cells with scattered Langerhans cells, giant cells, and large numbers of lymphocytes and plasma cells, indicating granulomatous inflammation. Small blood vessels showed fibrinoid necrosis with neutrophilic and lymphocytic infiltration. The pathological diagnosis was GPA. The immunohistochemical staining results were positive for CD-200 and CD-68. The rate of IgG4-positive staining was approximately 20% (Fig. 5b).

## Discussion and conclusions

The diagnosis of GPA is based on the 1990 ACR classification criteria. The sensitivity and specificity of these criteria are 88.2 and 92.0%, respectively. GPA mainly involves systems and organs with multiple small vessels, although the involvement of nearly every organ has been previously reported. The CNS is relatively rarely affected [2]. The manifestation of CNS involvement includes cranial nerve changes, meningoencephalitis, and infarction caused by angiitis [3]. The pituitary is very rarely involved. Until 2016, only 50 cases of GPA with pituitary involvement had been reported in the English literature (1 case series [4] and 42 single case reports [5, 6]). Among those 50 cases, all except one case, which was reported by G A Roberts in 1995, showed the involvement of other organs or systems [7]. The diagnosis of our patient was difficult during the progression of the disease because the isolated nature of the case did not match the ACR criteria; nevertheless, the pathological examination indicated GPA. Compared to previous cases, the unique characteristics of our case are its isolated nature and ANCA-negative status.

The first unique characteristic of our case is the isolated pituitary involvement. The only other similar case reported to date [7] occurred in an elderly woman who experienced onset with only ophthalmic symptoms, including decreased bilateral visual acuity with bilateral temporal hemianopsia and a pale nasal optic disc. This case was very similar to our case in terms of the MRI findings, endocrine test results and biopsy histopathology, as well as the several bouts of recurrence and surgical treatment (Additional file 1). The most significant difference between the case reported by Roberts and our case is the ANCA result. In the case reported by Roberts, the ANCA result was positive at onset and became negative after surgical treatment. Furthermore, in our case, the lesion was isolated in the anterior lobe of the pituitary, but in the case reported by Roberts, diabetes insipidus was present, indicating that both pituitary lobes were involved.

In other cases with pituitary involvement, lung and skin involvement reportedly occurred within 3 weeks [8] or several months [9, 10] after the first onset of diabetes insipidus or a decrease in pituitary hormone levels; pituitary dysfunction has also occurred during the development of the disease. However, in the present case, we observed the patient for more than 40 months after intense corticoid treatment, and no signs of the involvement of any other organs or systems were detected.

The second unique characteristic of our case is the ANCA negativity both at the first onset and during recurrence. Our literature review showed that the rate of ANCA-positive results is approximately 83–94% among all GPA cases and 91.9% among cases with pituitary involvement. This rate is related to the disease severity [11], and an ANCA test can be positive or negative at different points during the progression of the disease. The ANCA level appears to decrease or become negative during disease remission, and approximately 10% of GPA patients present with consistently negative results. Several GPA cases with isolated lung or kidney involvement have exhibited ANCA negativity, and our case is the first reported ANCA-negative case with isolated pituitary involvement. The relationship between ANCA negativity and isolated involvement is not clear. The number and function of CD25^hi^CD4^+^ cells are abnormal in GPA patients, and these cells are unable to inhibit the PR3 conversion of T lymphocytes.

We reviewed all 51 reported cases (including our case) for ophthalmic manifestations and found an ophthalmic involvement rate of 35% (20/51). Nine cases exhibited decreased visual acuity and VF defects. Two cases showed episcleritis; there were 2 cases of conjunctivitis, 1 case of dry eye and 2 cases of palsy of cranial nerves III and VI. Our patient presented with visual loss, VF defects, ptosis and abduction dysfunction. Compared to other pituitary lesions, GPA is more likely to involve more parts of the visual system, including the optic chiasm, optic nerve, cranial nerves III and VI, sclera and conjunctiva, and the progression of visual loss is more rapid because GPA is a form of vasculitis and is usually invasive. When the lesion spreads and invades or encircles the optic nerve, optic chiasm or cavernous sinus, severe visual loss with multiple cranial nerve palsies can occur within a very short time. Uveitis usually accompanies systemic vasculitis due to the abundant blood supply. Conjunctivitis and dry eye may be related to metabolic disorders caused by anterior pituitary dysfunction.

We analyzed the 9 cases that presented with visual loss and VF defects and found several similarities. First, 7 of 9 cases presented with both anterior lobe and posterior lobe dysfunction; 1 case presented with isolated anterior lobe dysfunction, and the adrenocortical pituitary hormone levels were not described in the remaining case. Anterior lobe involvement in these 9 cases was far greater than in all GPA cases (72%) [12], which means that there may be some links between visual loss and anterior lobe involvement. The common mechanism of visual loss caused by a pituitary lesion is the compression or invasion of the optic chiasm, which is related to the anatomical location and size of the lesion. The anterior lobe is located in the upper and frontal part of the pituitary and is the part of the pituitary closest to the optic chiasm. A lesion in that area usually has a suprasellar extension, which can easily lead to chiasmal compression. Second, in all 9 cases, other CNS lesions were near the sellar region and included meningitis and sphenoid and cavernous sinus enhancement. According to the literature, the rate of CNS involvement in pituitary GPA is approximately 42.5% [12], which suggests that when the optic chiasm is compressed or invaded, the lesions are more invasive.

The visual prognosis in some cases is extremely poor. Four of 9 patients presented with unilateral or bilateral blindness during disease development [13–15]. These four patients were relatively young. The visual losses all occurred suddenly during the first recurrence and decreased rapidly to NLP within several weeks. We documented the changes in the VF of our patient during this decrease and found that the changes in vision spread from the temporal section to the lower nasal section and then to the entire VF. The MRI scans all revealed enhancement of the suprasellar structure, including the optic chiasm and cavernous sinus, as well as hypothalamic infiltration. Three patients were healthy after treatment, and there was one death (Additional file 2).

In conclusion, we present a case of GPA with isolated pituitary involvement characterized by anterior lobe dysfunction, optic chiasm infiltration and ANCA negativity. Our review of the literature indicates that GPA with an ophthalmic manifestation is always caused by chiasmal invasion and is accompanied by anterior lobe dysfunction and other CNS lesions; furthermore, this type of case is more likely to be invasive. The onset of visual loss is usually rapid and severe during recurrence of the disease. In clinical work, the diagnosis of ANCA-negative GPA is usually difficult and relies solely on the pathological biopsy. Whether other organs will become involved or ANCA positivity will occur in this case remains to be seen.

## Additional files

Additional file 1: Comparison of 2 isolated GPA cases. In this file, we compared ophthalmological and MRI changes, pituitary function and prognosis of the isolated case of pituitary GPA reported by GA Roberts and our case. (DOCX 15 kb)

Additional file 2: Comparison of four GPA cases with bilateral visual loss. In this file, we compared ophthalmological and MRI changes, pituitary function and treatment of 4 GPA cases with bilateral visual loss reported in literature. (DOCX 21 kb)

## Abbreviations

ACR: American College of Rheumatology
ACTH: Adrenocorticotropic hormone
ANAs: Antinuclear antibodies
ANCA: Anti-neutrophil cytoplasmic antibody
BCVA: Best-corrected visual acuity
BDG test: 1,3-beta-D-glucan assay
CF: Counting fingers
CNS: Central nervous system
Cr: Creatinine
CRP: C-reactive protein
CSF: Cerebral spinal fluid
CT: Computed tomography
E2: Estradiol
ENA: Anti-extractable nuclear antigen
ESR: Erythrocyte sedimentation rate
FSH: Follicle-stimulating hormone
fT4: Free thyroxine
GC: Glucocorticoids
GPA: Granulomatosis with polyangiitis
H&E: Hematoxylin and eosin
HM: Hand motion
IOP: Intraocular pressure
IV: Intravenous
LH: Luteinizing hormone
MPO: Myeloperoxidase
MRI: Magnetic resonance imaging
NLP: No light perception
OCT: Optical coherence tomography
PR3: Proteinase 3
PRL: Prolactin
RAPD: Relative afferent pupillary defect
RF: Rheumatoid factor
TSH: Thyroid-stimulating hormone
T-SPOT test: Interferon gamma release assay for tubuerculosis
VF: Visual field
WBC: White blood cell
WG: Wegener’s granulomatosis
