# Supplementary material for: Severe ophthalmic manifestation in pituitary-involved granulomatosis with polyangiitis: a case report and literature review
Source: BMC Ophthalmol. 2018 Nov 16;18:299. doi: 10.1186/s12886-018-0966-0 (PMC6240177; doi:10.1186/s12886-018-0966-0)
Supplement: Supplementary file 1 — Comparison of 2 isolated GPA cases. In this file, we compared ophthalmological and MRI changes, pituitary function and prognosis of the isolated case of pituitary GPA reported by GA Roberts and our case. (DOCX 15 kb) [file 12886_2018_966_MOESM1_ESM.docx]

Additional file 1 Comparison of 2 isolated GPA cases

|  | Roberts [7] | Zhang |
| --- | --- | --- |
| **Age** | 71 | 20 |
| **Sex** | Female | Male |
| **Symptoms at onset** | Visual loss | Visual loss  Headache |
| **Visual field** | Bilateral hemianopsia | Bilateral hemianopsia |
| **MRI** | Large intrasellar mass with suprasellar extension, but no invasion of the cavernous sinus.  Recurrence: not mentioned. | Enlarged pituitary gland with increased T2 signal, heterogeneous enhancement, optic chiasm compression, and bilateral extension to the cavernous sinus and the suprasellar region |
| **Pituitary anterior lobe function** | Decreased TSH, fT4, fT3, cortisol, FSH, and LH; normal PRL | Decreased TSH, fT4, fT3, and E2; normal cortisol, FSH, LH, and PRL |
| **Pituitary posterior lobe function** | Diabetes insipidus after surgery | Normal |
| **ESR** | Normal | Normal |
| **CRP** | Elevated | Normal |
| **c-ANCA** | Positive | Positive |
| **Symptoms at recurrence** | Bilateral visual loss | Bilateral visual loss; headache |
| **Relapse period** | 2-4 months | 1 year |
| **Number of relapses** | 2 | 1 |
| **Pathology** | Connective tissue with slight inflammatory cell infiltration consisting predominantly of polymorphonuclear cells with some lymphocytes and occasional plasma cells | Normal arrangement of acinar cells with scattered Langerhans cells, giant cells, and a large number of lymphocytes and plasma cells; fibrinoid necrosis with neutrophilic and lymphocytic infiltration in the small vessels |
| **Follow-up time** | 21 months | 40 months |
