# Supplementary material for: Severe ophthalmic manifestation in pituitary-involved granulomatosis with polyangiitis: a case report and literature review
Source: BMC Ophthalmol. 2018 Nov 16;18:299. doi: 10.1186/s12886-018-0966-0 (PMC6240177; doi:10.1186/s12886-018-0966-0)
Supplement: Supplementary file 2 — Comparison of four GPA cases with bilateral visual loss. In this file, we compared ophthalmological and MRI changes, pituitary function and treatment of 4 GPA cases with bilateral visual loss reported in literature. (DOCX 21 kb) [file 12886_2018_966_MOESM2_ESM.docx]

Additional file 2 Comparison of four GPA cases with bilateral visual loss

| **Case** | McIntyre[13] | Lohr[14] | Hugues[15] | Zhang |
| --- | --- | --- | --- | --- |
| **Onset age** | 22 | 19 | 30 | 20 |
| **Sex** | Female | Female | Female | Male |
| **Onset symptoms** | Amenorrhea; headache | Nasosinusitis;  headache | NR | Visual loss;  headache |
| **Visual acuity prognosis** | Sudden bilateral blindness; no recovery after i.v. GC treatment | Bilateral blindness in 2 hr; left eye recovery after i.v. GC treatment | Bilateral blindness | Bilateral blindness;  no recovery after i.v. GC treatment |
| **Cranial nerve** | N III palsy | Diplopia | NR | N III, N IV palsy |
| **MRI** | Infiltration of the cavernous sinus, chiasm, and frontal lobe meninges | CT: thickening of the nasal mucosa | Infiltration of the hypothalamus, suprasellar region, cavernous sinus and chiasm | Infiltration of the cavernous sinus, chiasm, and frontal lobe meninges |
| **Function of adenohypophysis** | Decreased TSH, fT, FSH, LH, PRL, and E2 | Decreased function | Decreased TSH, FSH, and LH | Decreased TSH, fT4, and fT3 |
| **Function of neurohypophysis** | Diabetes insipidus after second surgery | Normal | Diabetes insipidus | Normal |
| **c-ANCA** | Negative; positive at recurrence | Negative | NR | Negative |
| **Relapse period** | Several days, 6 months | 1 year | NR | 1 year |
| Number of relapses | 2 | 1 | NR | 1 |
| Pathology | Optic nerve: chronic inflammatory process and Langerhans cells; pituitary: dense fibrous infiltrate with a few nests of nonspecific chronic inflammatory cells (1^st^ operation); necrotizing infiltrative inflammatory reaction with no staining for S100 or CD1a (2^nd^ operation) | Atypical cells | NR | Fibrinoid necrosis with neutrophilic and lymphocytic infiltration in the small vessels |
| Treatment | A trans-sphenoidal surgery and a trans-frontal craniotomy after the 1^st^ onset, followed by external beam radiotherapy and high-dose IV methylprednisolone and cyclophosphamide therapy | 2 operations after the 1st onset;  anterior pituitary hormone replacement therapy, GC and cyclophosphamide after recurrence | NR | Low-dose IV steroid after the 1^st^ onset; craniotomy biopsy followed by IV GC |
| Prognosis | Death within 13 months | 54 months | NR | 40 months |
